# Insights on multimorbidity and associated health service use and costs from three population-based studies of older adults in Ontario with diabetes, dementia and stroke

**DOI:** 10.1186/s12913-019-4149-3

**Published:** 2019-05-16

**Authors:** Lauren E. Griffith, Andrea Gruneir, Kathryn Fisher, Dilzayn Panjwani, Amiram Gafni, Christopher Patterson, Maureen Markle-Reid, Jenny Ploeg

**Affiliations:** 1 Department of Health Research Methods, Evidence, and Impact, McMaster University, McMaster Innovation Park, 175 Longwood Road South, Hamilton, ON L8P 0A1 Canada; 2 Department of Family Medicine, University of Alberta, 6-10 University Terrace, Edmonton, AB T6G 2T4 Canada; 3 School of Nursing, McMaster University, 1280 Main Street West, Health Sciences Centre, Room 3N25G, Hamilton, Ontario L8S 4K1 Canada; 4 Women’s College Research Institute, Women’s College Hospital, 790 Bay St., 7th floor, Toronto, ON M5G 1N8 Canada; 5 Centre for Health Economics and Policy Analysis; Department of Health Research Methods, Evidence, and Impact, McMaster University, 1280 Main Street West, Room CRL-208, Hamilton, Ontario L8S 4K1 Canada; 6 Department of Medicine, McMaster University, St. Peter’s Hospital, 88 Maplewood, Hamilton, Ontario L8M 1W9 Canada; 7 School of Nursing, McMaster University, 1280 Main Street West, Health Sciences Centre, Room 3N25B, Hamilton, Ontario L8S 4K1 Canada; 8 School of Nursing, McMaster University, 1280 Main Street West, Health Sciences Centre, Room 3N25C, Hamilton, Ontario L8S 4K1 Canada

**Keywords:** Multimorbidity, Comorbidity, Community-living older adults, Health service utilization, Health service costs

## Abstract

**Background:**

Most studies that examine comorbidity and its impact on health service utilization focus on a single index-condition and are published in disease-specific journals, which limit opportunities to identify patterns across conditions/disciplines. These comparisons are further complicated by the impact of using different study designs, multimorbidity definitions and data sources. The aim of this paper is to share insights on multimorbidity and associated health services use and costs by reflecting on the common patterns across 3 parallel studies in distinct disease cohorts (diabetes, dementia, and stroke) that used the same study design and were conducted in the same health jurisdiction over the same time period.

**Methods:**

We present findings that lend to broader Insights regarding multimorbidity based on the relationship between comorbidity and health service use and costs seen across three distinct disease cohorts. These cohorts were originally created using multiple linked administrative databases to identify community-dwelling residents of Ontario, Canada with one of diabetes, dementia, or stroke in 2008 and each was followed for health service use and associated costs.

**Results:**

We identified 376,434 indviduals wtih diabetes, 95,399 wtih dementia, and 29,671 with stroke. Four broad insights were identified from considering the similarity in comorbidity, utilization and cost patterns across the three cohorts: 1) the most prevalent comorbidity types were hypertension and arthritis, which accounted for over 75% of comorbidity in each cohort; 2) overall utilization increased consistently with the number of comorbidities, with the vast majority of services attributed to comorbidity rather than the index conditions; 3) the biggest driver of costs for those with lower levels of comorbidity was community-based care, e.g., home care, GP visits, but at higher levels of comorbidity the driver was acute care services; 4) service-specific comorbidity and age patterns were consistent across the three cohorts.

**Conclusions:**

Despite the differences in population demographics and prevalence of the three index conditions, there are common patterns with respect to comorbidity, utilization, and costs. These common patterns may illustrate underlying needs of people with multimorbidity that are often obscured in literature that is still single disease-focused.

**Electronic supplementary material:**

The online version of this article (10.1186/s12913-019-4149-3) contains supplementary material, which is available to authorized users.

## Background

Multimorbidity, i.e., the co-existence of 2 or more chronic conditions in the same person [1], is increasingly recognized as a significant driver of health service use and costs and is a challenge to care provision [2, 3]. Multimorbidity is common, especially in older adults. In a 2013 study including over 31 million Medicare beneficiaries, Salive et al. reported that overall 67% had multimorbidity; including 81.5% of those 85 years or older [4]. Although guidelines for the management of multimorbidity are now available [5], it has been noted that treating patients with multiple conditions is clinically challenging as many guidelines are still organized around single conditions [6–9]. More recently, however, there has been a call for a more patient-focussed approach [10] that has catalyzed the development and testing of multimorbidity-focussed interventions [11].

The authors are members of the Aging, Community and Health Research Unit (ACHRU) at McMaster University which studies index conditions within the context of comorbidity, with a particular focus on three vascular or vascular-related conditions: diabetes, dementia, and stroke [12]. While these 3 conditions differ in prevalence, presentation, and disease course, they were targeted because they all require self-management, systems navigation, and interprofessonal collaboration, which are key components of the community-based interventions developed by ACHRU. Moreover, the conditions that ACHRU researchers focus on are costly at a population level due to prevalence, intensity of care requirements, and shared risk factors that lead to a clustering of comorbidities. For example, diabetes is one of the top three chronic conditions in older adults and is also a major risk factor for cardiovascular disease [13], and cardiovascular disease, which includes stroke, is one of the leading causes of hospitalization and death in Canada [14]. In preparation for the development and testing of the ACHRU interventions, members of the research team carried out 3 separate cohort studies using population-based administrative data to characterize older adults living in the community with dementia, diabetes, or stroke in terms of their comorbidity profiles, selected demographic characteristics, and health service use and costs over a 5-year period.

Each of the cohort-specific findings is presented in three separte publications [15–17]. The tendency for authors, like ourselves, to publish these types of studies in specialty-specific journals, however, makes it difficult for researchers and clinicians from different disciplines to glean the broader insights into multimorbidity. This issue is further complicated by methodological differences across disciplines and studies that challenge further comparisons. The aim of this paper is to share insights on multimorbidity and the associated health services use and costs by reflecting on common patterns seen across the 3 parallel studies conducted in three distinct disease cohorts. Since all three studies pertain to the same time period, geographical area and health care system, we are able to control for some of the key factors that have been barriers to drawing more general insights from different cohorts in the past.

## Methods

The full methods for each of the original 3 retrospective cohort studies are presented in previous publications [15–17] and summarized in Additional file 1 In the following subsections, we provide a summary of those methods with an emphasis on the methods used for this paper relative to the original publications.

### Setting and data

The original studies were conducted on populations residing in Ontario, Canada’s most populous province with approximately 13 million residents. The provincial government is the sole insurer for medically necessary physician and hospital services through the provincial health insurance plan (OHIP), with over 98% of all physician expenditures and over 93% of all hospital expenditures being publicly financed [18]. As such the majority of health services used by Ontarians are captured in administrative data holdings. All Ontarians who are not covered by a federal reimbursement plan (e.g., persons living on reserves and other Aboriginal settlements, full-time members of the Canadian Forces, persons living in federal prisons) are not included in the administrative databases used in these studies. The administrative data holdings used in the three studies include the following services: physician visits, emergency department visits, hospital admissions, and home care. Outpatient prescription drug coverage was also available for those 65 years and older. The databases were linked using unique identifers. The data were analyzed at ICES which is a not-for-profit research institute in Toronto, Ontario. The data holdings at ICES are used extensively for research and to inform policy [19–23].

### Study cohorts

All community-living people in Ontario, aged 66 years and older and having an existing diagnosis of: 1) diabetes, 2) dementia, or 3) stroke as of April 1, 2008 (baseline) were included in the three cohorts. Because the 3 cohorts were originally analyzed separately, if a person had more than one of the index conditions, for example, diabetes and dementia, they would be included in both cohorts. Because we defined a “chronic condition” as one which lasted 6 months or longer [24], we examined only administrative data prior to October 2007 to determine disease status. We further exluded people ≥105 years of age, residing in a province other than Ontario, having no recorded health service use in the 5 year prior to baseline, and those receiving palliative care. Finally, we also excluded people living in long-term care because their patterns of health care utilization differ from older adults living in the community. A person was included until they either were admitted to long-term care, died, moved out of the province, or reached the end of the 5-year follow-up period. The same inclusion and exclusion criteria were applied to all 3 cohorts.

### Chronic conditions

In addition to the 3 index conditions used to create the original cohorts, the following conditions were identified: anxiety/depression, arthritis, cancer, chronic obstructive pulmonary disease (COPD), upper gastrointestinal bleed, hypertension, ischemic heart disease, liver disease, osteoporosis/osteopenia, inflammatory bowel disease, renal disease (with and without chronic dialysis), and cerebrovascular disease other than stroke. We defined each chronic condition using one of two methods. The first method used information on diagnostic codes from any of the administrative data holdings in the 5 year prior to baseline (i.e., 2008). The second method was based on entry into a a disease-specific database previously created at ICES (Additional file 2). To estimate comorbidity for each cohort, we summed the total number of the listed conditions, including the other 2 index conditions (e.g., for the diabetes cohort, we counted dementia and stroke in the total number of comorbid conditions).

### Health services utilization

Health service utilization included physician visits, emergency department visits, hospitalizations and home care contacts. For each cohort, we identified health services used for the index condition (i.e., diabetes, dementia, and stroke) and those used for other (non-index) conditions. For home care services we include all service types for the cost analysis, but focused on nursing visits for utilization, as they represent the majority of home care encounters. For home care services we were not able to distinguish between services used for index and non-index conditions.

### Cost calculations

To calculate costs we multiplied the published unit cost for each service by the volume of service (Additional file 3). We summed over all costs (total health service costs) as well as calculating the proportion of the total attributed to each of the service types: physician visits, emergency department visits, hospitalizations, and home care. In the original studies we examined utilization and costs over 5 years. Because we found similar trends over time and the focus of this paper is on the patterns across the 3 cohorts, for simplicity we present 1 year utilization and costs from the baseline date (2008).

### Statistical methods

Insights were drawn by the research team after reviewing and reflecting on the findings across the three cohorts. We present a number of key descriptive analyses from the original publications to support an understanding of the general insights. We focused on descriptive analyses in order to characterize the cohorts and their use of health services. For each cohort we describe the baseline distribution of age, sex, the number and type of comorbid chronic conditions, health service utilization, and costs. We also present health service utilization attributed to the index and non-index conditions and the percent of health service use costs attributed to each service type by the number of comorbid conditions. Finally we examined the unique impact of age and number of comorbid conditions on different types of health services.

### Ethics

The original studies were approved by the Research Ethics Boards at McMaster University and Sunnybrook Health Sciences Centre (Ethics certificate #: 13–394-C).

## Results

### Cohort characteristics and health services utilization

In Table 1, we present a brief summary of the demographic characteristics of each index cohort and health service utilization and costs over the first year. Complete details can be found in the original publications [15–17]. We identified 376,367 individuals who met the inclusion criteria for the diabetes cohort, 100,630 for the dementia cohort, and 29,673 for the stroke cohort (cohorts were not mutually exclusive). Among community-living adults aged 65 years and older in Ontario, this represented a prevalence of 22, 6, and 2% respectively for diabetes, dementia and stroke. The dementia and stroke cohorts were older on average (average age 80.9 and 78.5 year respectively) than the diabetes cohort (average age 75.3 years). Compared to the stroke and diabetes cohorts, the dementia cohort had more women than men. The prevalence of having 2 or more comorbid conditions (in addition to the index condition) was high in all cohorts at 76% in the diabetes cohort, 83% in the dementia cohort and 92% of the stroke cohort. In general, the members of the three cohorts saw a GP and specialist over once a month on average with the exception of GP visits by members of diabetes cohort which was an average of 10.4 visits/year. The average per-person health service use cost (in 2008 Canadian dollars) for the year was $3741 for people with diabetes, $7092 for people with dementia and $7786 for people with stroke.

**Table 1:** Socio-demographic, Comorbidity, and Health Care Utilization Characteristics of Diabetes, Dementia, Stroke Cohorts

	Diabetes	Dementia	Stroke
Total Cohort - 2008	376,421	100,630	29,673
Age groups: 66–74 years	189,867 (50.4%)	20,379 (20.3%)	9,471 (31.9%)
75–84 years	149,558 (39.7%)	47,937 (47.6%)	13,646 (46.0%)
85+ years	36,966 (9.9%)	32,314 (32.1%)	6,556 (22.1%)
Age – Mean (SD)	75.3 (6.5)	80.9 (7.2)	78.5 (7.2)
Gender: Male	189,005 (50.2%)	39,666 (39.4%)	14,817 (49.9%)
Female	187,416 (49.8%)	60,694 (60.6%)	14,856 (50.1%)
# of comorbid conditions: 0	16,515 (4.4%)	3,750 (3.7%)	255 (0.9%)
1	72,380 (19.2%)	13,415 (13.3%)	2,026 (6.8%)
2	113,893 (30.3%)	23,543 (23.4%)	5,162 (17.4%)
3	87,874 (23.3%)	23,923 (23.8%)	6,876 (23.2%)
4	49,615 (13.2%)	17,462 (17.4%)	6,295 (21.2%)
5+	36,144 (9.6%)	18,537 (18.4%)	9,059 (30.5%)
Average # comorbidities – Mean (SD)	2.5 (1.33)	2.9 (1.48)	3.5 (1.28)
Health Service Utilization 2008 – Mean (SD)			
General Practitioner Visits	10.38 (10.81)	12.81 (14.97)	13.24 (14.91)
Specialist Visits	12.89 (14.48)	13.47 (15.82)	15.64 (17.01)
Emergency Department Visits	1.80 (11.00)	1.65 (9.00)	2.40 (12.58)
Hospitalizations			
Medical	0.20 (0.61)	0.30 (0.71)	0.36 (0.82)
Surgical	0.08 (0.30)	0.07 (0.29)	0.09 (0.32)
ALC	0.03 (0.19)	0.08 (0.30)	0.07 (0.28)
ICU	0.04 (0.24)	0.04 (0.21)	0.06 (0.27)
Homecare nursing visits	2.96 (17.54)	3.95 (20.29)	4.32 (20.63)
Total Health Service Use Costs (2008)	$1,408,188,080	$713,692,137	$231,045,259
Average Costs Per Patient (2008)	$3,741	$7,092	$7,786

### Insight 1: Most common comorbid conditions

**Fig. 1:**
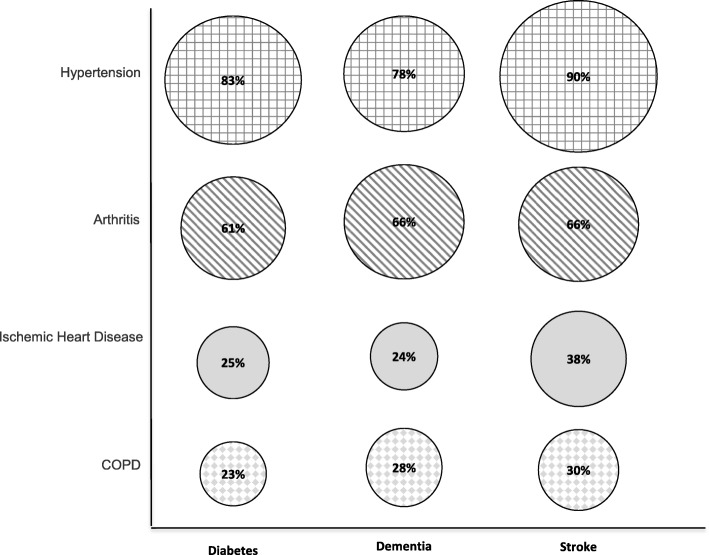
Prevalence of Most Common Comorbidities in the Diabetes, Dementia, and Stroke Cohorts

Figure 1 shows the prevalence of the four most common comorbid conditions for each index condition. Hypertension and arthritis were by far the dominant comorbid conditions in all three cohorts, with at least 78 and 61% of patients in the cohorts having hypertension and arthritis, respectively. The next most common conditions were far less prevalent, including ischaemic heart disease (38% or less) and COPD (30% or less).

### Insight 2: non-index condition-related service use

**Fig. 2:**
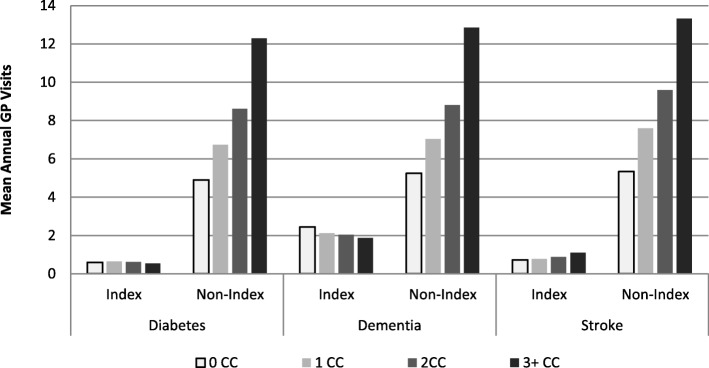
Average Number of General Practitioner Visits for Index and Non-Index Conditions by Number of Comorbid Conditions (CC) in the Diabetes, Dementia, and Stroke Cohorts

Across the three index-conditions, the vast majority of service utilization was attributed to non-index condition reasons. Figure 2 shows average number of GP visits attributed to care for the index and non-index conditions. The bulk of GP services for all three cohorts were for care that was attributed to non-index conditions. This was even the case where no other comorbid conditions were identified. These patterns were consistent across the three cohorts and for all service types.

### Insight 3: drivers of cost

**Fig. 3:**
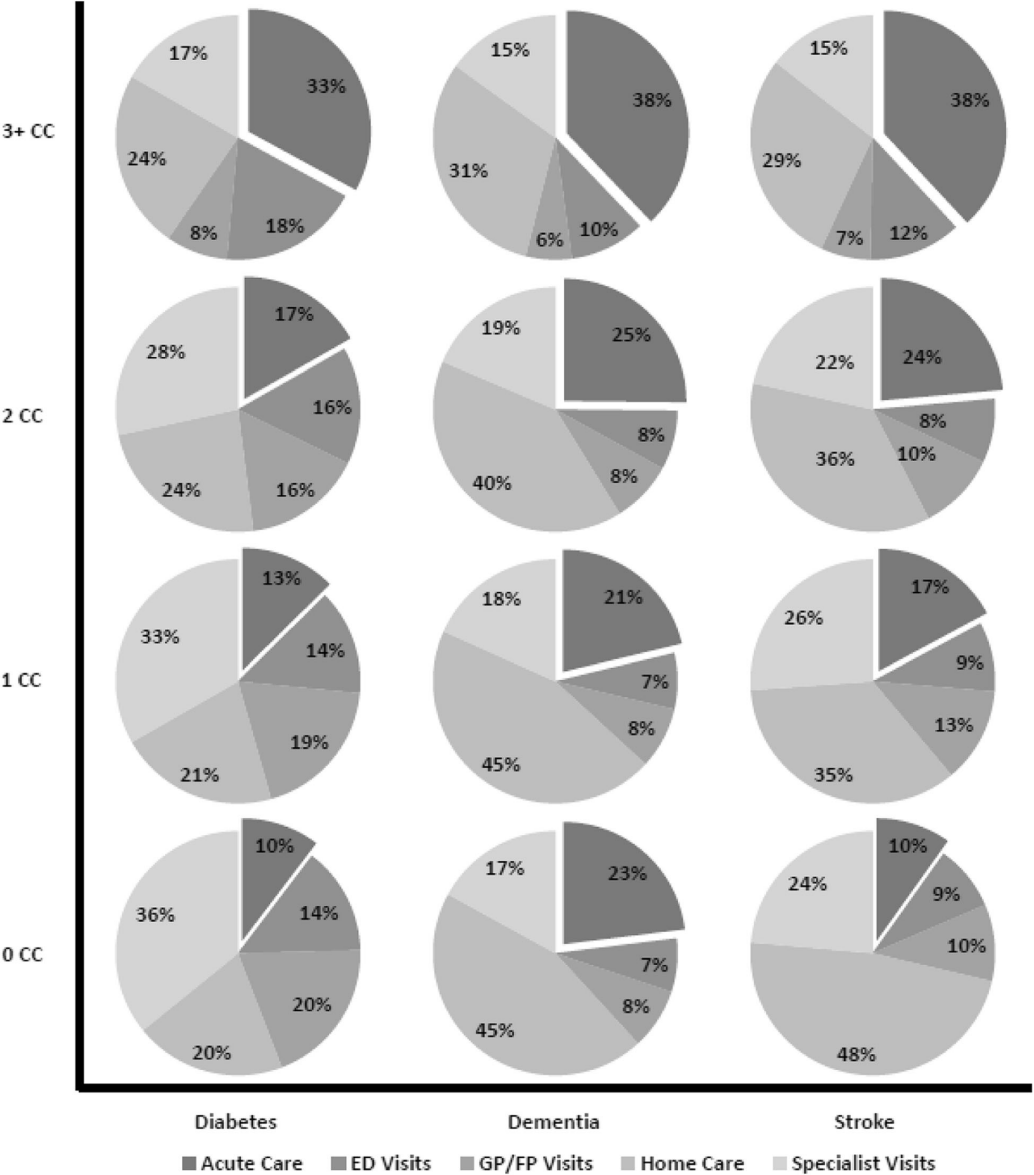
Percent of total health service use costs attributed to each service type: Acute Care, ED Visits, General Practitioner (GP)/Family Practioner (FP), Home Care, and Specialist Visits by the number of comorbid conditions (CC) in the Diabetes, Dementia, and Stroke Cohorts

Figure 3 displays the relative drivers of costs (GP, specialist, ED, inpatient acute care and home care) for each index condition cohort by the level of comorbidity. Among those with 0–1 comorbidities, the largest cost driver was community-based care, either home care or general physician services, whereas among those with higher comorbidity, acute care services became the primary cost driver. This was consistent across all three cohorts.

### Insight 4: age vs comorbidity

**Fig. 4:**
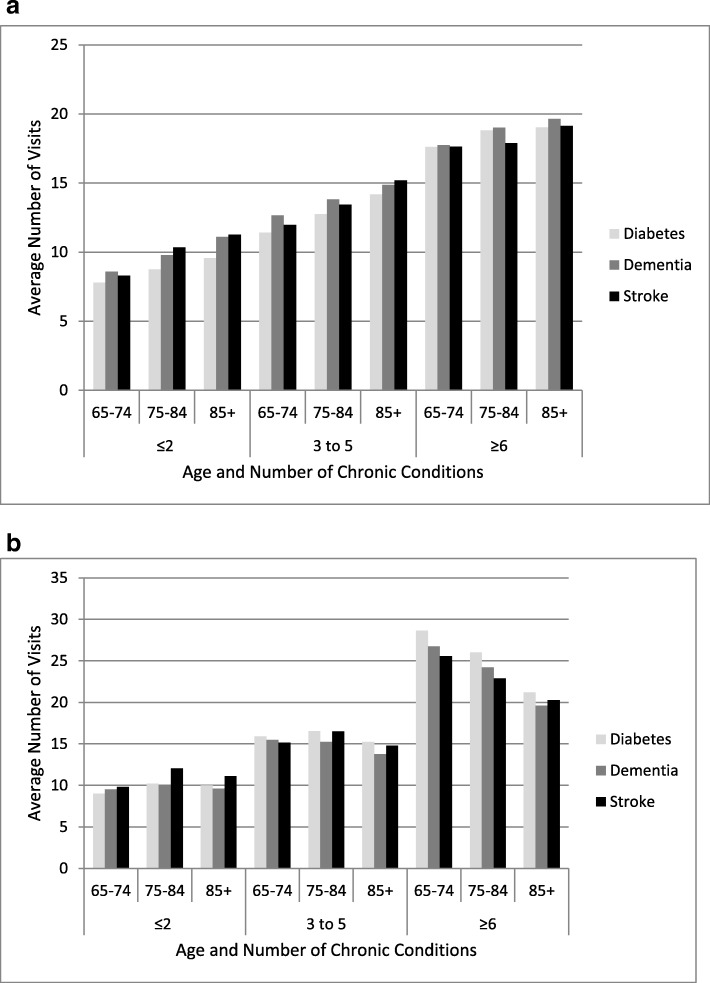
**a-b** Average Number of: **a** General Practioner Visits, and **b** Specialist Visits by Age and Number of Chronic Conditions in the Diabetes, Dementia, and Stroke Cohorts

Across all service types a higher level of comorbidity was associated with higher levels of utilization. Age-related patterns, which were similar across the three cohorts, differed across service types. Figure 4a and b demonstrate the consistency of the pattern among the cohorts by displaying the average number of GP and specialist visits by level of comorbidity and age. For each index condition, there is a clear increase in the average number of GP visits with both age and level of comorbidity (Fig. 4a). For specialist visits, there is a consistent increase with level of comorbidity, but the relationship with age is more complex despite similarities across the cohorts (Fiagure 4b). The age-related patterns do not appear to relate to characteristics of the cohort, but may relate to other issues such as characteristics of the service (e.g., service type, barriers to access).

## Discussion

The terms “comorbidity” and “multimorbidity” both refer to the occurrence of multiple chronic conditions within the same individual; however “comorbidity” refers to the effects of additional conditions in reference to an index chronic condition [25] (such as diabetes, dementia, and stroke) whereas the term “multimorbidity” indicates that no single condition holds priority over any of the co-occurring conditions [26]. Researchers and clinicians working in specific disease areas usually know the comorbidity literature that pertains to their area, whereas the multimorbidity literature may be less well known and can be unclear as to its applicability to their specific patients or practice settings [27]. Most of the literature on the burden and complexity of comorbidity in the context of an index condition is published in specialty-specific journals that are typically not easily synthesized across specialties. Drawing general inferences from multimorbidity literature is further complicated by the use of different study designs, multimoribidty definitions, and data sources [18]. In this paper we draw general insights about multimorbidity by observing patterns of comorbidity and associated health care utilization and costs from three community-based cohorts with three distinct index conditions [15–17]. By considering the common findings from these three cohorts, which were obtained utilizing similar methods and drawn from the same geographic population, we can identify key insights on multimorbidity that may be useful for practice, policy, and research across disciplines.

The three original studies, which focused on cohorts defined by having dementia, diabetes, and stroke, were conducted to support the development and implementation of RCTs to test interventions to improve community-based care among older adults living with at least one of those index conditions. The three conditions are relatively common in older adults, require monitoring and self-care and interprofessional collaboration between primary and secondary care. While all three conditions are vascular or vascular-related diseases, they differ significantly in terms of their prevalence, presentation, and disease course. Stroke is an acute event with a high mortality rate and variable longer term effects/needs including a range of physical and cognitive impairments and intensive treatment around the initial event followed by secondary stroke prevention [28]. Diabetes often appears earlier but progresses slowly with a range of effective treatments (e.g., lifestyle modifications, drug therapies) [29]. Dementia typically appears later in life but shows progressive decline with a limited and variable course and currently has no effective treatments that can significantly delay decline [30]. Despite these differences in the three conditions, there were a number of similarities in the patterns of comorbidity, health care utilization, and associated costs.

Our first insight was that hypertension and arthritis were by far the most common co-morbid conditions in all three cohorts. This finding is consistent with the literature, which shows that these two conditions alone or in combination are highly prevalent in many older adult populations [31]. In fact, in each of our disease cohorts over 75% of the population had either arthritis, hypertension, or both. This finding has implications for clinical care, which typically follows a one-condition-at-at-time approach and thus may result in treatment plans that do not consider the impact of comorbidities or plans that reduce the risks/symptoms for one condition yet increase them for another condition [1]. For example, Nonsteroidal Anti-Inflammatory Drugs (NSAIDs), a common treatment for osteoarthritis, can reduce the efficiency of antihypertensive drugs and may increase risk of cardiovascular disease and renal failure in people with hypertension [32, 33]. Osteoarthritis itself can also impact the care of other chronic conditions, especially those requiring self-management. In 2015, the Global Burden of Disease Consortium ranked osteoarthritis the 13th leading cause of disability from a total of 310 diseases and injuries [34] and indicated that this was largely due to associated pain [35]. Comorbid osteoarthritis has been shown to reduce quality of life in people with diabetes [36], prolong rehabilitation in stroke survivors [37], and amplify the association of other diseases with poor self-reported physical health [38]. More generally, individuals with a rheumatic disease and multimorbidity experience more impaired daily functioning and lower health related quality of life [39], both of which can impede self-management. This underscores the importance of considering arthritis management in any strategy to manage multiple chronic conditions in older adults [40].

Our second insight was that health service use in all three cohorts increased consistently and dramatically with the number of chronic conditions, and that the majority of health service use was not directly related to the index condition. This finding is consistent with the published literature [41]. In our study, among those who did not have any of the 14 comorbid conditions, the average number of non-index GP visits was 2–3 times higher than GP visits for the index condition. This may in part result from specific chronic conditions that are missing in our comorbidity list, however our list does include the most common chronic conditions in Canada [42] as well as those typically included in multimorbidity research [43]. It could also reflect the under-diagnosis of chronic conditions in older adults [44] or other social determinants of health on patterns of health care utilization [45]. These findings underscore the complexity of multimorbidity and how it represents something more than a simple tally of chronic conditions. In terms of research, this also implies that overall health care utilization may be a better way to evaluate the impact of interventions rather than disease-specific health care utilization even when considering interventions targeted toward specific chronic conditions.

The third insight is that the share of health care costs attributed to inpatient acute care increased as the number of conditions increased. Across the three cohorts, home care, GP visits, and specialist visits accounted for the majority of costs, but were differently distributed among those without other comorbid conditions. For example, home care represented 20% of the costs for the people with diabetes alone and 48% of the costs for people with stroke alone. As the level of comorbidity increased, the distribution of costs began to look more similar across disease cohort, with at least one-third attributed to acute care across each cohort. Although we are unable to comment on the reasons for, or the appropriateness of, this increasing acute care use with greater comorbidity does speak to the potential for community-based programs and services to better support people living with complex conditions to prevent the need for potentially avoidable and costly acute care episodes.

Our fourth insight was that the comorbidity and age-related patterns of health service use were the same across the cohorts yet differed across services. Muggah et al. (2012) [46] show similar findings in terms of both comorbidity and service use and age-related differences in service use for GP versus specialist visits. These findings suggest that characteristics of the services and/or access to them may be important in understanding service use in relation to comorbidity and age. Additionally, the chronic conditions and socio-demographic factors we considered may not include all of the reasons older adults seek healthcare services. For example, older adults may have health issues such as incontinence and pain that are not readily captured in a limited list of medical diagnoses. It is also possible that the pattern reflects the focus of specialists’ care. It may be, for example, that diabetologists and endocrinologists would want to see patients with diabetes more frequently at an earlier age when there is more opportunity to treat aggressively in the hope of reducing later morbidity and mortality. Teasing out the potential underlying factors driving service use requires further study across a broader range of services, disease cohorts, and socio-demographic characteristics.

Overall the patterns that we found across the three index conditions in terms of comorbidity, utilization and costs reflect the medical complexity of these patients and underscore the need for management programs/interventions to shift from a single disease-centred approach to a focus on multimorbidity. This is not to say that interventions should focus only on multimorbidity. A 2016 systematic review of interventions to manage patients with multimorbidity in primary care and community settings found that interventions targeted either at specific combinations of common conditions or at specific problems for patients with multiple conditions, may be more effective than a more general approach [47]. The review authors also concluded that organizational interventions, such as the introduction of the clinical nurse to support the treatment of depression were more effective than organizational interventions that had a broader focus, such as case management or changes in care delivery for all individuals with multimorbidity. This suggests that although multimorbidity is a core issue, practical solutions may need to focus on individual conditions or risk factors while incorporating strategies to manage the common features of multimorbidity. Whatever the focus, the patterns we found with respect to comorbidity and its impact on health service use and costs seem to persist across the three cohorts and need to be on the radar when considering how to best manage complex patients with multimorbidity and informing policy and decision makers on health care planning in the context of multimorbidity.

Using comprehensive administrative data, our three original studies included all community-living older adults in Ontario with diabetes, dementia and stroke in 2008. Since all three studies pertain to the same time period, geographical area and health care system, we are able to control for some of the key factors that have been barriers to drawing more general insights from different cohorts in the past. However, our original studies did have limitations. First, we considered only health services that were covered by the public health insurance system, which would not capture privately-paid home care services. We also included only 14 potential comorbidities whereas many comorbidity lists are much more extensive. Although this may be a limitation, it should be noted that we included the most common chronic conditions in Canadians [42] and those most commonly used multimorbidity research [43]. However, the list does not include things like pain, physical functioning, or cognitive changes that may be the actual reason that a person goes to the doctor or seeks services. And even among those conditions included in the list, conditions such as depression and anxiety are known to be under-represented in administrative databases and are common drivers of health service use [48]. We also did not include informal care in our cost and utilization estimates, which can be significant in older adults with diabetes, dementia and stroke survivors [49–51]. Finally, we used only the most responsible condition listed in the administrative data to classify health services into those attributable to the index-condition and those attributable to non-index-conditions, however, a consistent trend was found over all types of health services as well as different administrative data sources.

## Conclusions

Despite the underlying differences in the biological processes of the 3 index conditions and demographics of the study cohorts, common patterns of comorbidity and health service use emerged which supports viewing these results with a multimorbidity lens. While the limitations of single disease frameworks to meet the needs of a population increasingly characterized by multiple chronic conditions have long been recognized, how to better address multimorbidity in care planning is still unclear. Our findings reflect population-level burden which helps to highlight some key issues that may be useful for better service planning. Future studies could use more complex regression methods to examine the effect of demographic and socioeconomic variables on the relationship between multimorbidity and health services utilization which could provide further context for multimorbidity research as well as identify subgroups who may benefit most from multimorbidity interventions.

## Additional files

Additional file 1: Details of Study Methods for 3 Cohort Studies (Diabetes, Dementia, and Stroke). (DOCX 35 kb)

Additional file 2: Diagnostic Definitions for Index Conditions (Diabetes, Dementia, Stroke) and Co-morbid Conditions. (DOCX 22 kb)

Additional file 3: Costing Methods and Data Sources. (DOCX 15 kb)

## Abbreviations

ACHRU: Aging, Community and Health Research Unit
CAD: Canadian dollar
CC: Chronic condition
CIHI: Canadian Institute for Health Information
CIHR: Canadian Institutes of Health Research
COPD: Chronic obstructive pulmonary disease
ED: Emergency department
GP: General practitioner
MHLTC: Ministry of Health and Long-Term Care
OHIP: Ontario Health Insurance Plan
RCT: Randomized Controlled Trial
