# Supplementary material for: Insights on multimorbidity and associated health service use and costs from three population-based studies of older adults in Ontario with diabetes, dementia and stroke
Source: BMC Health Serv Res. 2019 May 16;19:313. doi: 10.1186/s12913-019-4149-3 (PMC6524233; doi:10.1186/s12913-019-4149-3)
Supplement: Supplementary file 1 — Details of Study Methods for 3 Cohort Studies (Diabetes, Dementia, and Stroke). (DOCX 35 kb) [file 12913_2019_4149_MOESM1_ESM.docx]

Supplementary Appendix 1: Details of Study Methods for 3 Cohort Studies (Diabetes, Dementia, and Stroke)

Study Design

A retrospective cohort study design was used for each cohort including multiple linked administrative databases to examine health service utilization and costs of a population-based cohort of community-living older adults with 1) diabetes, 2) dementia, and 3) stroke.

Setting

Each study used data from Ontario Canada which has approximately 13 million residents. The vast majority of Ontarians are covered under the provincial health insurance plan (OHIP). Coverage includes outpatient physician visits, acute care hospital use (both in the emergency department and inpatient admission), homecare, and outpatient prescription drug coverage for those 65 years and older. In Ontario, Community Care Access Centres provide publicly funded homecare, using a contractual model of service delivery, wherein publicly funded case managers contract out homecare services to community agencies that provide care to clients.

Data

The administrative databases linked in this study include: the Registered Persons Database (RPDB) for basic demographic data on all individuals enrolled in the provincial insurance program; the OHIP claims database for physician visits; the Discharge Abstract Database (DAD) for all records of inpatient hospitalizations; the National Ambulatory Care Reporting System (NACRS) for all records of emergency department visits and other ambulatory contacts; the Same Day Surgery (SDS) database for same-day surgeries and procedures, the Home Care Database (HCD) for information on all homecare service records; and the Ontario Drug Benefits (ODB) claims database for all outpatient prescription claims. Additional data sources were accessed to obtain specific diagnostic information. These included the Ontario Mental Health Reporting System (OMHRS), the Ontario Cancer Registry (OCR), and the Ontario Diabetes Database (ODD). The data were linked using unique, encoded identifiers and analyzed at the Institute for Clinical Evaluative Sciences (ICES) in Toronto, Ontario. These data are regularly used for research purposes and have been studied extensively for their validity.[1-6]

Study Cohorts

The three cohorts consists of all individuals aged 66 and over, who resided in the community, and had an existing diagnosis of: 1) diabetes, 2) dementia, and 3) Stroke as of April 1, 2008 (baseline). We set our lower age limit at age 66 to have at least one year of available prescription claims, which was necessary to identify some chronic conditions. The information used to identify the three cohorts is summarized in Supplementary Appendix 2. We identified people diagnosed with diabetes within the 5 years prior to baseline using the Ontario Diabetes Database (ODD). We defined dementia as the presence of at least 1 diagnostic code in the OHIP claims within the 5 years prior to baseline, or 1 International Classification of Disease (either version 9 or 10 depending on year) in DAD or NACRS within the 5 years prior to baseline, or 1 claim in the ODB for a cholinesterase inhibitor in the year prior to baseline. This definition of dementia has been used in other population-based studies that have used administrative databases.[7] We looked for evidence of stroke within the 5 years prior to baseline by searching all diagnostic fields within the DAD for any relevant International Classification of Disease version 10 (ICD-10-CA) codes. The list of ICD-10-CA codes is based on those used in a recent evaluation of stroke care in Ontario.[8] Ischemic and hemorrhagic strokes were both included. For each cohort we required that any relevant claims or codes be identified prior to October 2007 to ensure that individuals had the condition for at least 6 months at baseline, which was part of our definition of a chronic condition.[9] Individuals who were 105 years or older, receiving palliative care (in any setting), residing outside of Ontario, or had no contact with the health system in the five years prior to the baseline date were excluded. We also excluded individuals residing in long-term care homes since they tend to have different patterns of health service utilization than community-living older adults. From baseline, each individual was followed until the first of: admission into a long-term care home, death, a move out of province, or the end of the 5-year follow-up period.

Comorbid Conditions

The comorbid chronic conditions identified were: anxiety/depression, arthritis, cancer, chronic obstructive pulmonary disease (COPD), upper gastrointestinal bleed, hypertension, ischemic heart disease, liver disease, osteoporosis/osteopenia, inflammatory bowel disease, renal disease (with and without chronic dialysis), and other cerebrovascular disease. The list was chosen to utilize pre-existing validated ICES disease algorithms and registries. Each condition was defined using either of the following methods: 1) a search for diagnostic codes in any of the OHIP, DAD, or NACRS databases and/or specification prescription claims within the 5 years prior to baseline; or 2) entry into a diagnosis-specific database created at ICES. (Supplementary Appendix 2) To estimate comorbidity for each cohort, we summed the total number of the listed conditions, including the other 2 index conditions (e.g.,, for the diabetes cohort, we counted dementia and stroke in the total number of comorbid conditions) (0, 1, 2, or 3 or more).

Health Services Utilization

We identified all publically-funded health services utilization during the study period. This included physician visits (both primary care and specialist), unplanned emergency department visits, hospitalizations, and homecare contacts. We identified hospitalization “episodes” so that transfers between hospitals were not counted as separate events. For each hospitalization episode, we estimated the total length of stay (admission to final discharge), the total number of days in the intensive care unit (ICU), and total number of days designated as Alternate Level of Care (ALC). ALC refers to periods of time when a patient is considered to no longer require hospital-level care but cannot be discharged due to a lack of alternative services (for example, when a long-term care bed is required but not available). This is particularly relevant in dementia patients since it is the most frequent diagnosis associated with ALC designation.[10] We distinguished physician visits, emergency department visits, and hospitalizations for each of diabetes, dementia, and stroke (index condition) from those for all other reasons (non-index conditions). For homecare services, we counted the total number of visits and specific visit types (including case management, nursing, in-home support, and therapies) for cost analyses, but focused on nursing visits for utilization as they are the most common type of service. We were unable to distinguish between homecare services for index and non-index reasons.

Statistical Methods

We described each cohort at baseline by age, sex, neighbourhood income quintile, and the number and type of comorbid chronic conditions. For each year of follow-up, we estimated the total amount of each type of health service use (physician services, acute care, and homecare) by baseline comorbidity status. Costs for each type of health service were calculated by multiplying the volume of service (either total number of visits or hours of service, depending on type) by the published unit cost (cost per visit or hour, depending on type).(Supplementary Appendix 3). Total service costs for each year in the 5-year follow-up were calculated by adding the total costs per type. Average annual per patient costs were estimated by dividing total service costs by the number of individuals in the cohort at the beginning of the year. We also estimated the proportion of total health service costs attributed to physician visits, emergency department visits, hospitalizations, and homecare. All costs in the original 3 cohort papers were expressed in 2012 dollars. Because we found similar trends over time in the original studies and the focus of this paper is on the patterns across the 3 cohorts, for simplicity in this paper we focus on 1 year utilization and costs from the baseline date (2008) and reported the results in 2008 dollars. Finally, because the main intent of this study was to present descriptive statistics on the full population of community-living older adults with 1) diabetes, 2) dementia, and 3) stroke in Ontario, the decision was made not to use statistical tests given the large cohort size which would have resulted in small p-values.

Reference List

1. Gruneir A, Bell CM, Bronskill SE, Schull M, Anderson GM, Rochon PA: **Frequency and pattern of emergency department visits by long-term care residents--a population-based study.** *J Am Geriatr Soc* 2010, **58:** 510-517.

2. Gruneir A, Bronskill S, Bell C, Gill S, Schull M, Ma X *et al*.: **Recent health care transitions and emergency department use by chronic long term care residents: a population-based cohort study.** *Journal of the American Medical Directors Association* 2012, **13:** 202-206.

3. Rochon PA, Bronskill SE, Gruneir A, Liu B, Johns A, Lo AT *et al*.. Ontario Women's Health Equity Report. Older Women's Health. Edited by Rochon PA. 2011. Toronto, St. Michael's Hospital and the Institute for Clinical Evaluative Sciences.

4. Hux JE, Ivis F, Flintoft V, Bica A: **Diabetes in Ontario: determination of prevalence and incidence using a validated administrative data algorithm.** *Diabetes Care* 2002, **25:** 512-516.

5. Vermeulen MJ, Tu JV, Schull MJ: **ICD-10 adaptations of the Ontario acute myocardial infarction mortality prediction rules performed as well as the original versions.** *Journal of Clinical Epidemiology* 2007, **60:** 971-974.

6. Hawes C, Morris JN, Phillips CD, Mor V, Fries BE, Nonemaker S: **Reliability estimates for the Minimum Data Set for nursing home resident assessment and care screening (MDS).** *Gerontologist* 1995, **35:** 172-178.

7. Seitz DP, Gill SS, Gruneir A, Austin PC, Anderson GM, Bell CM *et al*.: **Effects of dementia on postoperative outcomes of older adults with hip fractures: a population-based study.** *J Am Med Dir Assoc* 2014, **15:** 334-341.

8. Hall R, Khan F, O'Callaghan C, Kapral MK, Hodwitz K, Fang J *et al*.. Ontario stroke evaluation report 2012: prescribing system solutions to improve stroke outcomes. Toronto: Institute for Clinical Evaluative Sciences . 2012.

9. O'Halloran J, Miller GC, Britt H: **Defining chronic conditions for primary care with ICPC-2.** *Fam Pract* 2004, **21:** 381-386.

10. McCloskey R, Jarrett P, Stewart C, Nicholson P: **Alternate Level of Care Patients in Hospitals: What Does Dementia Have To Do With This?** *Can Geriatr J* 2014, **17:** 88-94.
