# Supplementary material for: Insights on multimorbidity and associated health service use and costs from three population-based studies of older adults in Ontario with diabetes, dementia and stroke
Source: BMC Health Serv Res. 2019 May 16;19:313. doi: 10.1186/s12913-019-4149-3 (PMC6524233; doi:10.1186/s12913-019-4149-3)
Supplement: Supplementary file 2 — Diagnostic Definitions for Index Conditions (Diabetes, Dementia, Stroke) and Co-morbid Conditions. (DOCX 22 kb) [file 12913_2019_4149_MOESM2_ESM.docx]

Supplementary Appendix 2: Diagnostic Definitions for Index Conditions (Diabetes, Dementia, Stroke) and Co-morbid Conditions

| Chronic Condition | Diagnostic Code |
| --- | --- |
| Diabetes  [Previous 5 years] | Ontario Diabetes Database, based on the following:  From CIHI-DAD or CIHI-SDS, one of the following:  ICD-9: 250  ICD-10: E10, E11, E13, E14  OR  From OHIP, 2 diagnostic codes within 2 years or 1 fee code:  Diagnostic code: 250  Fee code: Q040, K029, K045, K046 |
| Dementia  [Previous 5 years in DAD, OHIP, and previous 1 year ODB] | OHIP DXCODE: 290, 331, 797; OR  CIHI ICD-9: 290.0, 290.1, 290.3, 290.4, 290.8, 290.9, 294.1, 294.8, 294.9, 331.0, 331.1, 331.2, 797;  OR  CIHI ICD-10: F00.0, F00.1, F00.2, F00.9, F01.0, F01.1, F01.2, F01.3, F01.8, F01.9, F02.0, F02.1, F02.2, F02.3, F02.4, F02.8, F03, F05.1, F06.5, F06.6, F06.8, F06.9, F09, G30.0, G30.1, G30.8, G30.9, G31.0, G31.1, R54;  OR  ODB: A cholinesterase inhibitor (donepezil, galantamine, or rivastigmine) in the past 1 year  DIN: 02232043, 02232044, 02269457, 02269465, 02244298, 02244299, 02244300, 02244302, 02266717, 02266725 |
| Stroke  [Previous 5 years in DAD] | At least one hospitalization (DAD) for stroke – in any diagnosis field  G45 (excl. G45.4), H34.0, H34.1, I60 (excl. I60.8), I61, I63 (excl. I63.6), I64 |
| Anxiety and/or Depression  [Previous 5 years in DAD, OHIP, ODB and OMHRS] | One or more hospitalizations with a diagnosis for depressive disorder, affective psychoses, neurotic depression or adjustment reaction: ICD-10-CA codes F31, F32, F33, F341, F38.0, F38.1, F41.2, F43.1, F43.2, F43.8, F53.0, F93.0 or with a diagnosis for an anxiety state, phobic disorders or obsessive-compulsive disorders: ICD-10-CA codes F40, F41.0, F41.1, F41.3, F41.8, F41.9, F42, F45.2  OR  One or more hospitalizations with a diagnosis for anxiety disorders: ICD-10-CA codes F32, F341, F40, F41, F42, F44, F45.0, F45.1, F45.2, F48, F68.0, or F99 AND one or more prescriptions for an antidepressant or mood stabilizer: ATC codes N05AN01, N05BA, N06A  OR  One or more physician visits with a diagnosis for depressive disorder or affective psychoses: ICD-9-CM codes 296, 311  OR  One or more physician visits with a diagnosis for anxiety disorders: ICD-9-CM code 300 AND one or more prescriptions for an antidepressant or mood stabilizer: ATC codes N05AN01, N05BA, N06A  OR  Three or more physician visits with a diagnosis for anxiety disorders or adjustment reaction: ICD-9-CM code 300, 309  OMHRS: Section Q, item 1Mood/anxiety = yes if f or g = 1, 2, or 3 on discharge assessment  OR  Substance abuse/ addictions:  OMHRS Section Q, item 1 Mood/anxiety = yes if d= 1,2 or 3 |
| Arthritis  [Previous 5 years in DAD, NACRS, OHIP] | OHIP: 274, 446, 710, 711, 714, 715, 716, 718, 720, 727, 728, 729, 739  ICD-10: M05-M06, M15-M19, M07, M10, M11-M14, M30-36, M00-M03, M20-M25, M65-M79 |
| Inflammatory Bowel Disease  [Previous 5 years in DAD, NACRS and OHIP] | ICD10: K500, K501, K508-K515, K518, K519, M074, M075, M091, M092, K52  OHIP 555, 556, 564 |
| Cancer  [Since 1964] | Inclusion in Ontario Cancer Registry (OCR) |
| Chronic obstructive pulmonary disease [Previous 5 years] | From CIHI-DAD or CIHI-SDS, one of the following:  ICD-9: 491, 492, 496  ICD-10: J41, J42, J43, J44  OR  From OHIP, one of the following:  Diagnostic code: 491, 492, 496 |
| Congestive heart failure  [Previous 5 years ] | From CIHI-DAD, one of the following:  ICD-9: 428  ICD-10 I50  OR  From OHIP, 2 of the following within a one-year period:  Diagnostic code: 428 |
| Upper Gastrointestinal Bleed  [Previous 5 years in DAD] | ICD-10: K25.0, K25.2, K25.4, K25.6, K26.0, K26.2, K26.4, K26.6, K27.0, K27.2, K27.4, K27.6, K28.0, K28.2, K28.4, K28.6, K92.0, K92.1, K92.2 |
| Hypertension  [Previous 5 years ] | Ontario Hypertension Database: case-definition algorithm of 2 physician billing claims or 1 hospital discharge with a diagnosis of hypertension in a 2-year period that had the following diagnostic codes: I10.x, I11.x, I12.x, I13.x, or I15.x |
| Ischemic Heart Disease  [Previous 5 years in DAD and OHIP] | ICD-10: I20-I25  CCP: 4802, 4803, 4809, 481  CCI: 1IJ50, 1IJ57GQxx, , 1IJ76  Or having two physician billings within a one-year period with one of the billings by a specialist or a family physician in a hospital or emergency room setting) or a hospital discharge abstract.  OHIP: 410, 412, 413, R742, R743, Z434, G298 |
| Liver disease  [Previous 5 years in DAD and SDS] | Mild: ICD-10 codes (starting with) B18, K700-K703, K709, K713-K715, K717, K73, K74, K760, K762-K764, K768, K769, Z944  Moderate/Severe: ICD-10 codes (starting with) I850, I859, I864, I982,K704, K711, K721, K729, K765, K766, K767 |
| Osteoporosis/ Osteopenia  [Previous 5 years in DAD, SDS, NACRS, and ODB] | One of hip, wrist, spine, shoulder, or pelvis fracture OR osteoporosis treatment  Hip Fx: S72.0, S72.1, S72.2, S72.3  With external code:  W00--‐W10, W18 or W19  Wrist Fx: S52.x  W00--‐W10, W18 or W19  Spine Fx: S22.0, S22.1, S22.2, S22.3, S22.4, S32.0, S32.2, T08.xx  W00--‐W10, W18 or W19  Shoulder Fx: S42.2  W00--‐W10, W18 or W19  Pelvis:  S32.1, S32.3, S32.4, S32.5, S32.7, S32.8  W00--‐W10, W18 or W19  Must contain both an external cause of injury  code indicating fracture was due to a minor fall  AND a diagnosis code of fracture.  For inpatient and same day surgery records, the hip/wrist/spine/shoulder/pelvis fracture diagnosis must be the most responsible diagnosis. For ED records, the hip/wrist/spine/shoulder/pelvis fracture diagnosis must be one of the first 3 diagnoses (dx10code1 – dxcode3)  Exclude the fracture if the record contains any diagnosis code starting with‘V’ (motor vehicle accident). Exclude anyone who had a hospital diagnosis (any dxtype) of ICD-10 code: G40, G41 or cancer during the fiscal year of the fracture or during either of the two preceding fiscal years |
| Renal Disease  [Previous 5 years in DAD and OHIP] | Any 2 codes within 90 days of one another:  With Chronic Dialysis: OHIP: R849, R850, G323, G325, G326, G330, G331, G860, G333, G083, G091, G085, G295, G082, G090, G092, G093, G094, G861, G862, G863, G864, G865, G866, G294, G095, G096  CCP: 51.95, 66.98  CCI: 1PZ21HQBR, 1PZ21HPD4  Any of following ICD10 codes or OHIP codes:  Without Chronic Dialysis: dx10code= E102, E112, E132,E142,I12, I13, N08, N18, N19), or OHIP DXCODE in (403, 585) |
| Cerebrovascular disease (not stroke) [Previous 5 years in DAD and SDS] | G45, G46, H340, I60-I69 |

CIHI: Canadian Institute for Health Information

DAD: Discharge Abstract Database

DXCODE: Diagnostic code

ICD-9: International Classification of Disease, version 9

ICD-10: International Classification of Disease, version 10

ODB: Ontario Drug Benefit Plan

OHIP: Ontario Health Insurance Plan

OMHRS: Ontario Mental Health Reporting System

NACR: National Ambulatory Care Reporting System

SDS: Same Day Surgery (derived from NACRS)
