# Supplementary material for: Insights on multimorbidity and associated health service use and costs from three population-based studies of older adults in Ontario with diabetes, dementia and stroke
Source: BMC Health Serv Res. 2019 May 16;19:313. doi: 10.1186/s12913-019-4149-3 (PMC6524233; doi:10.1186/s12913-019-4149-3)
Supplement: Supplementary file 3 — Costing Methods and Data Sources. (DOCX 15 kb) [file 12913_2019_4149_MOESM3_ESM.docx]

**Supplementary Appendix 3: Costing Methods and Data Sources**

Costs were calculated by service type for each year in the 5-year follow up. In general, service costs were calculated by multiplying the total number of visits/hours of service (volume) by the cost per visit/hour (unit cost). The specific methods used for costing each service type, including the source and method of calculating unit costs, were as follows:

1. Physician Visits: Costs were calculated separately for the two groups (general practitioners/family physicians and specialists), with volumes multiplied by the unit cost for each physician group to determine total costs. Unit costs for the two physician groups were obtained from the OHIP Schedule of Benefits (the provincial physician billing database), and represent the average across the billing codes commonly used for the index condition (diabetes, dementia, stroke). Determination of the commonly-used billing codes was made in consultation with two primary care physicians.
2. Emergency Department Visits: Costs represent the sum of the costs for Emergency Department (unplanned) visits and costs of ambulance services pertaining to these visits. Costs of Emergency Department visits were determined by multiplying total (unplanned) visits by the average cost per visit, with the latter representing the total of the physician salary (OHIP Schedule of Benefits) and the provincial average cost of emergency department visits (MOHLTC Health Data Branch Web Portal, Health Indicators Tool, Ontario Cost Distribution Methodology, Data Comparison Report 3A, <https://hsimi.on.ca/hdbportal/>).
3. Costs for ambulance services relating to the visits were determined by multiplying the total number of ambulance rides by the province-wide standard reimbursement amount obtained from the OHIP Schedule of Benefits.
4. Hospitalizations: Costs were calculated separately for medical, surgical, ALC and ICU hospital episodes. For each type of episode, total costs were obtained from the product of the total number of episodes, the average length of stay for that episode, and the provincial average per diem rate. The average per diem for ICU episodes was calculated from per diem rates provided by the MOHLTC for all provincial hospitals (MOHLTC Data Request IMC-0000001044). The same average per diem rate was used for medical, surgical and ALC episodes (MOHLTC Health Data Branch Web Portal (Ontario Cost Distribution Methodology, Data Comparison Reports, 6a Acute Inpatient, <https://hsimi.on.ca/hdbportal/>).
5. Note that only 45% of total ALC costs were included in the acute care costs, representing the estimated proportion of patients in ALC *not* waiting for a placement in a palliative care or long term care facility (patients excluded from our study as discussed above) (<http://www.longwoods.com/content/20674/>).
6. Home Care Visits: Costs were calculated separately for each home care service, which included nursing, respiratory, nutrition, therapies (physiotherapy, occupational therapy, speech and language pathology, social work, psychology), case management and shift services (nursing, personal support, homemaking, combined homemaking and personal support). Costs by service type for all non-shift services were calculated by multiplying the total number of visits by the provincial average cost per visit (MOHLTC Health Data Branch Web Portal, Health Indicators Tool, CCAC Functional Centres, LHIN Comparison, <https://hsimi.on.ca/hdbportal/> ). Shift service volumes are measured in hours (rather than visits), and these costs were calculated by multiplying the total number of hours by the hourly rate applicable to the service (MOHLTC Health Data Branch Web Portal, Community Care Access Centres, Other Client Services Functional Centres, MIS Comparative Reports, Tables 11A and 19A, <https://hsimi.on.ca/hdbportal/>; HSPRN, Table 2 – Historical Unit Costs by Health Care Setting, <http://www.hsprn.ca/uploads/files/Guidelines_on_PersonLevel_Costing_May_2013.pdf>).

Total service costs for each year in the 5-year follow-up were calculated by adding up the total costs for each service type (calculated as described above). Per patient costs were calculated by dividing total service costs by the number of patients in the cohort, which declines in number over the 5 years as a result of patients moving to a location outside Ontario, death, or transfers out of the community to other care settings (e.g., long term care, palliative care).
